# Chimeric antigen receptor T-cell therapy for autoimmune diseases of the central nervous system: a systematic literature review

**DOI:** 10.1007/s00415-024-12642-4

**Published:** 2024-09-14

**Authors:** Agni M. Konitsioti, Harald Prüss, Sarah Laurent, Gereon R. Fink, Christoph Heesen, Clemens Warnke

**Affiliations:** 1 Department of Neurology, University of Cologne, Kerpener Str. 62, 50937 Cologne, Germany; 2 Department of Neurology and Experimental Neurology, Charité - Universitätsmedizin Berlin, Berlin, Germany; 3 Cognitive Neuroscience, Institute of Neuroscience and Medicine (INM3), Research Center Jülich, Jülich, Germany; 4 Department of Neurology, University Medical Center Hamburg-Eppendorf (UKE), Hamburg, Germany; 5 Faculty of Medicine, University Hospital Cologne, Cologne, Germany

**Keywords:** CAR T-cell therapy, Central nervous system autoimmunity, Antibody-mediated CNS disorders, Multiple sclerosis, NMOSD

## Abstract

**Importance:**

B-cell-targeting monoclonal antibodies have demonstrated safety and efficacy in multiple sclerosis or anti-aquaporin-4 IgG positive neuromyelitis optica spectrum disorder. However, these therapies do not facilitate drug-free remission, which may become possible with cell-based therapies, including chimeric antigen receptor (CAR) T cells. CAR T-cell therapy holds promise for addressing other antibody-mediated CNS disorders, e.g., MOG-associated disease or autoimmune encephalitis.

**Objective:**

To provide an overview of the current clinical knowledge on CAR T-cell therapy in central nervous system autoimmunity.

**Evidence review:**

We searched PubMed, Embase, Google Scholar, PsycINFO, and clinicaltrials.gov using the terms ‘CAR T cell’ and ‘multiple sclerosis/MS’ or ‘neuromyelitis optica/spectrum diseases/NMOSD’ or ‘MOG-associated disease/MOGAD ‘or’ autoimmune encephalitis’ or ‘neuroimmunology’.

**Findings:**

An ongoing phase I clinical trial has indicated the safety and benefits of anti-BCMA CAR T cells in 12 patients with AQP4-IgG seropositive neuromyelitis optica spectrum disorder. Case reports involving two individuals with progressive multiple sclerosis and one patient with stiff-person syndrome demonstrated a manageable safety profile following treatment with anti-CD19 CAR T cells. Recruitment has commenced for two larger studies in MS, and a phase I open-label basket study is underway to evaluate BCMA-directed CAR T cells in various antibody-associated inflammatory diseases, including MOG-associated disease. Preclinical research on NMDA receptor antibody autoimmune encephalitis treated with chimeric autoantibody receptor T cells generated promising data.

**Conclusions and relevance:**

There is minimal evidence of the benefits of CAR T-cell therapy in individuals with central nervous system-directed autoimmunity. Nevertheless, multicenter controlled clinical trials with a manageable safety profile appear feasible and are warranted due to very promising case experiences.

**Supplementary Information:**

The online version contains supplementary material available at 10.1007/s00415-024-12642-4.

## Introduction

### The role of the B-cell lineage in central nervous system autoimmunity

Autoreactive B cells play a crucial role in the pathogenesis of several autoimmune diseases [1]. Besides other antigen-presenting cells such as dendritic cells, B cells present self-peptides through the major histocompatibility complex, thereby activating autoreactive T cells [1, 2]**.** This collaboration of autoreactive T and B cells perpetuates chronic inflammation [3]. In addition, in autoimmune diseases where a pathogenic antibody defines the disease, B-cell-derived plasmablasts and plasma cells produce these autoantibodies [4]. Monoclonal antibodies (mAbs) targeting the B-cell lineage, initially developed for B-cell malignancies, have emerged as a widely utilized treatment modality for various autoimmune disorders. mAbs directed toward the CD20 antigen, such as rituximab [5, 6], ocrelizumab [7], ofatumumab [8], or ublituximab [9], have demonstrated a favorable safety profile and efficacy in multiple sclerosis (MS). More recently, in aquaporin-4 (AQP4) positive neuromyelitis optica spectrum disorder (NMOSD), the anti-CD19 antibody inebilizumab [10] as well as the IL6-receptor blocking agent satralizumab [11], which interferes with B-cell activation, have demonstrated efficacy. These studies in MS and NMSOD confirm the significant role of the B-cell lineage in central nervous system (CNS) autoimmunity. Although anti-CD20 mAbs have proven effective in reducing relapse rates and inflammatory activity in relapsing forms of MS, their impact on disability progression, as measured by the Expanded Disability Status Scale (EDSS), remains limited. Treatments for autoimmune encephalitis (AE) utilizing mAbs have become common practice [12], but have not received official approval. Similarly, for myelin oligodendrocyte glycoprotein antibody disease (MOGAD), approved therapies are yet to be developed.

### Limitations of B-cell-targeting monoclonal antibodies in treating CNS autoimmunity

mAbs targeting the B-cell lineage are designed as continuous therapies, without established de-escalation or cessation strategies making long-lasting, drug-free remission currently unrealistic. One explanation why continuous therapy is necessary is that—while peripheral circulating autoreactive B cells are effectively depleted by mAbs—their counterparts residing in remote lymphatic organs, such as the brain and the spinal cord, may evade the depletion [13]. TBX21-high memory B cells, which drive chronic inflammation, are primarily found in tissues close to the site of inflammation, exhibit a double-negative phenotype (CD19-negative, CD20-negative) enabling them to escape depletion [14]. Similarly, the primary source of autoantibodies in autoimmune diseases—whether from B-cell-derived plasmablasts or long-lived plasma cells—remains uncertain [15]. The lack of CD20 or CD19 expression on a proportion of long-lived plasma cells may allow them to evade current mAb therapies, thus potentially reducing their effectiveness. Additionally, the blood–brain barrier poses a significant physical barrier, complicating the effective biodistribution of mAbs within the CNS [16]. Other challenges, such as macrophage phagocytic defects, a scarcity of natural killer cells, and restricted vascular access, further hinder the ability of mAbs to induce antibody-dependent cellular phagocytosis and cytotoxicity [17].

Clinicians have been trying, e.g., by autologous hematopoietic stem cell transplantation (aHSCT), to develop strategies to reboot the immune system [18] to achieve prolonged disease remission and permanent discontinuation of immunosuppressive drugs. Although high rates of freedom of disease activity (clinical relapses and MRI activity) are observed in persons with MS following aHSCT, the evidence for progressive forms of MS remains less convincing [19, 20]. Besides, considerable possible risks and long-term toxicity of lymphoablative therapy, e.g., loss of ovarian function and infertility [21], treatment-related mortality [20, 22], and the occurrence of malignancies [23], may limit the broad use of aHSCT in CNS autoimmunity.

### CAR T cells and their use in non-CNS autoimmune disease and pre-clinical models

**Fig. 1:**
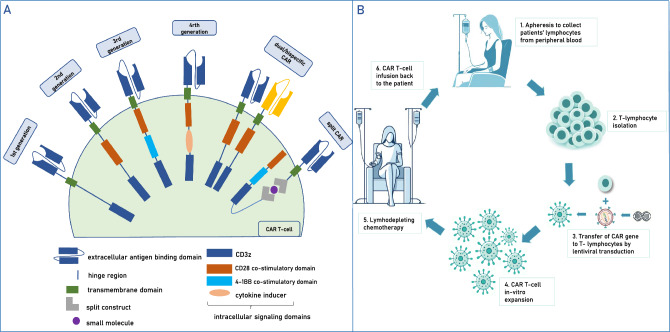
**A** CAR T-cell structure: first-generation CARs contain an intracellular signaling domain of CD3 zeta chain alone; second-generation CARs includes a single co-stimulatory domain (CD28 or 4-1BB); third-generation CARs combine two of the co-stimulatory domains; fourth generation CARs with transgene to express cytokines [48]; bispecific/dual-CAR T cells: CAR T cells recognizing two targets simultaneously on target cells; split signal CARs: The two split structures of the CAR-T cell are assembled and activated in the presence of a specific particular molecule. **B** CAR T cells—the application in clinical practice

CAR T-cell therapy targeting B cells has emerged as a revolutionary approach in the treatment of various malignancies. Its potential application in autoimmune diseases is currently under intense investigation and has entered earlier-stage clinical trials. For detailed information on the structure, antigen targets, clinical applications, and safety of CAR T cells, please refer to textbox 1 and Fig. 1.

Starting with rheumatic diseases, such as systemic lupus erythematosus (SLE) [24, 25], pemphigus vulgaris [26], systemic sclerosis [25], refractory antisynthetase syndrome [27, 28] and inflammatory myositis [29], a growing number of case reports and case series have demonstrated that anti-CD19 CAR T cells improve clinical scores, can reduce autoantibodies, and may achieve long-lasting remissions (most extended relapse-free remission 29 months for SLE cases [29]), making CAR T cells prime candidates for a superior intervention compared to standard treatments. A recent prospective open-label, non-randomized phase 1b/2a study [30] using mRNA-transduced CAR T cells targeting BCMA provided the first reassuring data for the treatment of refactory myasthenia gravis, and thus of a neuroimmunological autoimmune disease outside the CNS. In the same indication, case reports with CAR T cells targeting BCMA [31], CD19 [32], or bispecific CAR T cells targeting both CD19 and BCMA [33] have shown an association with decreases in all measures of disease severity, serologic remission, a favorable safety profile, and suppression of disease activity for up to 18 months [30, 34].

Unlike B-cell-targeting mAbs, CAR T cells are autonomous, self-amplifying effector cells and do not require natural killer cells, macrophages, or the complement system to perform [14]. Their ability to penetrate tissues, particularly the brain, and deplete otherwise inaccessible B cells that drive chronic, tissue-resident inflammation may explain their rapid and long-lasting therapeutic effects as reported so far in oncological [35] and SLE [29] patients.

Moreover, no long-term B-cell aplasia has been observed after CAR T-cell treatment. On the contrary, B cells generally reconstitute within 4–18 months after administration [29, 31, 33]. Analysis of the reconstituted B cells from peripheral blood has shown that preexisting memory B cells and plasmablasts disappear, indicating a switched naive B-cell phenotype and, therefore, a reboot of the B-cell compartment [31, 36]. Vaccination antibody titers were also found to be maintained in most reported cases with anti-CD19 CAR T-cell administration [24, 29, 32].

Regarding the potential application of CAR T cells for treating CNS autoimmunity, case reports document the successful use in CNS leukemia [37] and primary CNS lymphoma [38] with anti-CD19 CAR T cells. These reports highlight the CAR T cells’ favorable biodistribution properties to cross the blood–brain barrier and deeply deplete B cells in both the periphery and the remote CNS compartment. However, the substantially disrupted blood–brain barrier in CNS malignancies may facilitate the CAR T cells expansion, and it remains uncertain whether such expansion will be as effective in autoimmune diseases of the CNS.

**Fig. 2:**
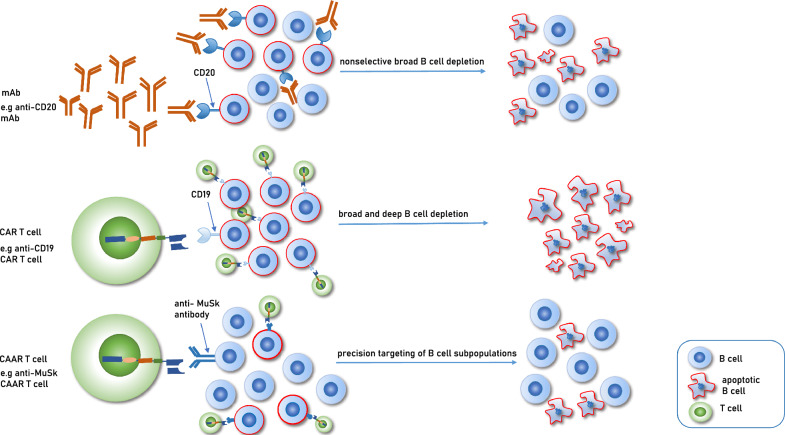
Mechanism of action of three B-cell depleting therapies. *mAb* monoclonal antibody, *CAR T* chimeric antigen receptor T cell, *CAAR T* chimeric autoantibody receptor T cell, *MuSK* muscle-specific kinase

While significant successes have been observed with anti-CD19 and anti-BCMA CAR T cells, these strategies result in broad B-cell depletion for several months. To avoid general immunosuppression, researchers are developing precision-targeted approaches for autoreactive B-cell subpopulations expressing autoantibodies on their surface. These T cells are genetically modified with a chimeric receptor containing the target antigen of the autoantibodies as an extracellular binding domain, known as chimeric autoantibody receptor (CAAR) T cells [39] (Fig. 2). In an experimental NMDAR AE model [40] and a myasthenia gravis mouse model [41], NMDAR CAAR T cells and MuSK CAAR T cells were administered. The data from the autoimmune myasthenia gravis mouse model contributed to an investigational new drug application and phase 1 clinical study design for the treatment of MuSK autoantibody-positive myasthenia gravis [41].

Textbox 1: CAR T cells—structure, antigen targets, application in clinical practice, and safety **CAR T cells—structure**

CAR T cells are autologous T cells and consist of four main components: (i) an extracellular target antigen-binding domain directed toward the desired target (mainly derived from an antibody fragment), (ii) a hinge region, (iii) a transmembrane domain, and (iv) one or several intracellular signaling domains, which activate CAR T cells after antigen binding to the extracellular domain [50] (Fig. 1A). This combination enables T-cell activation upon contact with the target cell antigen, bypassing antigen-presenting cells. The evolution of CAR from first to fourth generation includes the addition of co-stimulatory domains to assure proper expansion and activation of CAR T cells and target cell killing as well as co-expression of additional transgenes for cytokine secretion [48]. Recent advancements include the development of dual-targeting (bispecific) CAR T cells and split signal CARs, which become activated in the presence of a specific small molecule [51].

**CAR T cells—antigen targets for autoimmune disease**

CD19-directed CAR T-cell therapy is most widely used among various antigen targets. CD19 is a promising target as it is highly specific for the B-cell lineage and is expressed widely across different B-cell differentiation stages, including plasmablasts and a larger proportion of plasma cells (suppl. table 1) [52]. Another promising target is the B-cell maturation antigen (BCMA), primarily expressed on plasmablasts and plasma cells, including long-lived plasma cells. Bispecific CAR T cells that simultaneously target B cells through CD19 and plasmablasts through BCMA have recently been developed and applied to treat refractory myasthenia gravis [33].

**CAR T cells—their application in clinical practice**

Leukocytes are initially collected from peripheral blood through apheresis to manufacture CAR T cells. Subsequently, lymphocytes are transduced with a retro- or lentiviral vector encoding the CAR, followed by in vitro expansion. Alternative non-viral methods for gene transduction, e.g., with CRISPR-Cas9 gene editing, are also under investigation [53]. For optimal expansion and persistence of CAR T cells, lymphodepleting chemotherapy (typically cyclophosphamide and fludarabine) is administered before CAR T-cell infusion. The induced lymphopenia results in compensatory proliferation of CAR T cells and the formation of a new memory phenotype [54]. After lymphodepleting chemotherapy, CAR T cells are infused, where they further expand and eliminate the targeted B cells (Fig. 1B).

**CAR T cells—safety**

Conventional CAR T-cell engineering relies on DNA to express the CAR. The DNA is integrated permanently into the T-cell genome and replicates with each cell division [55]. This post-infusion proliferation of CAR T cells, sometimes referred to as a “living drug”, can lead to unpredictable pharmacokinetics and characteristic adverse events, such as cytokine release syndrome (CRS) and immune effector cell-associated neurotoxicity syndrome (ICANS) [56]. In oncology, ICANS typically manifests a few days following the onset of CRS and is observed in 20–60% of patients treated with either anti-CD19 or anti-BCMA CAR T cells [57]. Early symptoms include dysgraphia, speech impairment, tremor, cognitive impairment, and fatigue, which require consistent monitoring. In more severe cases, epileptic seizures, increased intracranial pressure, and even coma can occur [14]. The pathophysiology of this condition is not well understood, but there is evidence that endothelial activation and disruption of the blood–brain barrier are involved [36]. CRS rates, including milder forms of the condition, range from around 40–90% across all therapeutic cell products, with 10–30% of patients developing severe (grade > 2) CRS, which can lead to life-threatening and lethal events [58]. Treatment of CRS and ICANS includes antipyretics, glucocorticoids, and IL-6 receptor blockade with tocilizumab [59]. Thus far, CAR T-cell-mediated toxicity was less frequently observed in autoimmune diseases compared to hematological malignancies. A likely reason is the substantially lower quantity of targeted B cells in autoimmune disorders compared to B-cell-derived malignancies [60]. To address the unregulated CAR T-cell proliferation, transient mRNA-based CAR T-cell therapy has recently emerged as an alternative approach and has already been tested in refractory MG cases [30], delivering CAR-encoding mRNA into T cells without permanently altering their genomes. This approach allows for the temporal expression of CARs without the risk of genotoxicity and long-term elimination of specific cell types. The mRNA CAR-activity is, however, only temporary, necessitating repeated administration (see supplementary Fig. 2). Furthermore, the impact of the CAR-encoding mRNA on the risk of CRS is less certain as the CRS is also influenced by various characteristics of the host and the target cells.

## Aim and methods

Overall, considering the limited effects of approved mAbs on disease progression in MS discussed above, the putative requirement for life-long therapy currently without a de-escalation strategy despite cumulating risks, and the toxicity of rescue therapies such as aHSCT, it is warranted to explore newer cell-based therapies. Owing the promising findings of the use of CAR T cells in non-CNS autoimmune disease and pre-clinical models of CNS autoimmune disease, in this review, we aim to provide a comprehensive overview of current research on CAR T-cell therapies for refractory, progressive or relapsing immune-mediated diseases of the CNS, including MS, NMOSD, MOGAD, and some subentities of AE. We will address the challenges and prospects of targeting compartmentalized immunity in the brain and spinal cord and discuss upcoming CAR T-cell clinical trial programs for these conditions.

The systematic review was conducted according to PRISMA guidelines. We searched PubMed, Embase, Google Scholar, and PsycINFO for articles published from December 2016 to July 2024, using the search terms ‘CAR T cell’ and ‘multiple sclerosis/MS’ or ‘neuromyelitis optica/spectrum diseases/NMOSD’ or ‘mog-associated disease/MOGAD ‘ or ‘autoimmune encephalitis’ or “neuroimmunology”. Also, we screened clinicaltrials.gov for registered clinical trials using the same terms. We restricted our search to articles published in English. The search was done by one reviewer (AK), checked by a second reviewer (CW). We included pre-clinical and clinical studies, mechanistic studies, case reports, case series, and reviews on CAR T-cell therapy into our references. Articles cited in the studies or review articles were also considered.

## Results

Fifty-four articles and twelve registered clinical trials were screened and assessed for eligibility. After exclusion of articles not closely related to the topic and after removing of duplicates (PRISMA flow diagram, suppl. Figure 1), 19 articles and 9 clinical trials were selected for inclusion. Among these, four articles focused on the use of CAR-T cells in experimental autoimmune encephalomyelitis (EAE) or MS, three in NMOSD, and two in AE or an animal model of AE. Additionally, ten articles discussed the use of CAR T cells in neuroimmunology in general. We identified two ongoing clinical trials on NMOSD, four on MS, and three basket trials including patients with various autoimmune diseases, such as MS, MOGAD, and NMOSD. For details on the rationale for using CAR T cells in MS, NMOSD, MOGAD, and AE, please see textbox 2. A summary of registered clinical trials and case reports using different CAR T cells for treating neurological autoimmune diseases of the CNS is shown in Table 1, including the number of participants per study.

**Table 1:** (a) Registered clinical trials and case reports using different CAR T-cell types for the treatment of autoimmune diseases of the CNS (b)Published results from clinical trials and case reports using different CAR T cell types for the treatment of autoimmune diseases of the CNS

Registered clinical trials and case reports using different CAR T-cell types for the treatment of neurological autoimmune diseases of the CNS							
Title	Participants	Indication	NCT Number	Location	Status	Target antigen	Primary endpoints
NMOSD							
Treatment of Relapsed and/or Refractory AQP4-IgG Seropositive NMOSD by Tandem CAR T Cells Targeting CD19 and CD20, phase I study^b^	No information provided	AQP4-IgG seropositive NMOSD	NCT03605238	Beijing, Beijing, China	Withdrawn (recruitment failure)	CD19 and CD20	- Occurrence of AEs from baseline to 12 months post-infusion
Evaluate the Safety and Efficacy of CAR-T Cells in the Treatment of R/R NMOSD, phase I, single-arm, open-label, single-center study^b^	Estimated Enrollment: *n* = 9	Relapsed and/or Refractory NMOSD	NCT05828212	Hangzhou, Zhejiang, China	Recruiting	CD19	- DLT from baseline to 28 days post- infusion - AE and SAE from admission to end of follow-up, up to 2 years - Maximum tolerable dose
MS							
Study of KYV-101, an Autologous Fully Human Anti-CD19 CAR T-cell Therapy, in Subjects with Refractory Primary and Secondary Progressive MS (KYSA-7), phase II, open-label, randomized, multicenter study^a^	Estimated Enrollment: *n* = 120	Primary and secondary progressive MS	NCT06384976	Location not provided	Not yet recruiting	CD19	Efficacy of KYV-101 from baseline to 12 months post-infusion by rating disability on the EDSS scale
A Study to Evaluate the Safety, Tolerability, Efficacy, and Drug Levels of CC-97540 in Participants with Relapsing or Progressive Forms of MS, phase I, multicenter, single-arm study^b^	Estimated Enrollment: *n* = 98	Relapsing and progressive MS	NCT06220201	United States, Belgium, France, Germany, Italy, Spain, United Kingdom	Recruiting	CD19^A^	Up to week 104 post-infusion: - Number of participants with AEs, SAEs and AESIs - Number of participants with laboratory test result abnormalities - Number of participants with imaging abnormalities - Number of participants with DLTs - Recommended Phase 2 dose based on the incidence of DLTs
A Study of Anti-CD19 CAR T-cell Therapy in Subjects with Non-relapsing and Progressive Forms of MS, a phase I, open-Label, single Center Study^b^	Estimated Enrollment: *n* = 12	Progressive MS	NCT06138132	Palo Alto, California, United States	Recruiting	CD19	Frequency of DLT at each dose level up to 12 months post-infusion
A Study of KYV-101, a CD19 CAR T Cell Therapy, in Participants with Treatment Refractory Progressive Multiple Sclerosis, phase I, open-label, Single Center Study^b^	Estimated Enrollment: *n* = 10	Progressive MS	NCT06451159	University of California, San Francisco, United States	Recruiting	CD19	- The presence of CAR-T cells in CSF following their peak expansion in peripheral blood up to 48 weeks post-infusion - Incidence and severity of AEs and DLTs up to 48 weeks post-infusion
Basket trials							
A Study of C-CAR168 in the Treatment of Autoimmune Diseases Refractory to Standard Therapy, phase I, multicenter, open-label study^b^	Estimated Enrollment: *n* = 30	NMOSD RRMS Mediated Necrotizing Myopathy SLE	NCT06249438	Shanghai, Shanghai, China	Recruiting	CD20 and BCMA	- Incidence of AEs throughout the first 24 months post-infusion - Recommended dose of C-CAR168 based on DLTs rates and overall safety profile
Safety and Efficacy of CT103A Cells for Relapsed/Refractory Antibody-associated Inflammatory Diseases of the Nervous System, early phase I, open-label study^b^	Estimated Enrollment: *n* = 36	MS, AQP4-IgG seropositive NMOSD, MOGAD, AE, CIDP, IIM, MG, POEMS Syndrome	NCT04561557	Wuhan, Hubei, China	Recruiting, first results published (see below)	BCMA	- DLT from baseline to 3 months post-infusion - Incidence and severity of AEs up to 2 years post-infusion
Universal CAR-T Cells in Patients with Refractory Autoimmune Diseases of the Nervous System, early phase I, open-label, Single Center Study^b^	Estimated Enrollment: *n* = 25	MS, NMOSD, MG, CIDP	NCT06485232	Beijing, China	Not yet recruiting	CD19 and BCMA	- Incidence of DLTs the first 28 days after infusion - Incidence of AEs and SAEs up to 12 months after infusion

Published results							
Title	Participants	Indication	Target antigen	Primary endpoints	Exploratory outcomes	Adverse events	Laboratory results
Clinical trials							
Anti-BCMA CAR T-cell therapy (CT103A) in relapsed or refractory AQP4-IgG seropositive neuromyelitis optica spectrum disorders: phase I trial interim results, CARTinNS, 01/2023, from trial NCT04561557,^b^ [46]	*n* = 12	AQP4-IgG seropositive NMOSD	BCMA	- DLT after CT103A cells infusion up to 3 months post-infusion - Incidence and severity of AEs up to 2 years post-infusion	- No relapse reported in 11 of 12 patients (92%) at 5.5 months. - All corticosteroids and immunosuppressants were discontinued. - Disabilities and quality-of-life outcomes improved in all patients	- Grade 1–2 CRS reported in all patients. - Grade 3 or higher AEs occurred including neutropenia (*n* = 12), anemia (*n* = 6), and thrombocytopenia (*n* = 3). - Infections (*n* = 7), including 3 with serious CMV infections and 1 with pneumonia	- Serum AQP4-IgG for all participants decreased after infusion, and was negative by 12 weeks in 7/10 patients (70%), and by 6 months in 5/6 patients (83%). - Serum BCMA levels reduced below the lower limit of detection within 1-month post-infusion and returned to baseline levels by 6 months. - Significant decrease in total serum immunoglobin after infusion in all patients
Case studies							
CD19-targeted chimeric antigen receptor T-cell therapy in two patients with MS, Hamburg, Germany, 01/2024,^e^ [44]	*n* = 2	MS	CD19	–	- Pat. 1: EDSS score increased to 6.0 before returning to baseline (4.5) by day 29 and remaining stable. Walking distance increased from 400 m at baseline to 700 m at day 100 post-infusion. - Pat. 2: EDSS remained stable. - Other neurological assessments including timed 25-ft walk, 9-hole peg test, and 6-min walking distance matched pre-infusion scores in both patients	- Pat. 1: CRS grade 1, with facial and neck swelling and transient increase in transaminases. - Pat. 2: transient increase of transaminases (CTCAE grade 3) which improved with ursodeoxycholic acid. - No other adverse events occurred	- Pat. 1: OCBs decreased from 13 to 6 on day 14 after infusion and were sustained through day 64. - Pat. 2: Number of OCBs and intrathecal immunoglobulin levels remained stable - The residual B-cell population of patient 1 was depleted by day 2 post-infusion and has not reconstituted as of day 100. B-cell population of Pat. 2 was already depleted prior to CAR T-cell infusion. - Reduced serum immunoglobulin G was observed in both patients at day 14 post-infusion
Successful use of anti-CD19 CAR T cells in severe treatment-refractory stiff-person syndrome, Germany, 06/2024,^e^ [47]	*n* = 1	SPS	CD19	–	- Reduced pain and leg stiffness in a 5-month follow-up. - Walking speed increase over 100%. - Daily walking distance improvement from < 50 m to > 6 km within 3 months - GABAergic medication (benzodiazepines) was reduced by 40%. - No immunotherapy was administered post-CAR T cell infusion	- Grade 2 CRS with fever (maximum of 38.3 °C) and transient hypotension successfully treated with paracetamol, dexamethasone, and tocilizumab. - Sore throat and cervical lymph node swelling were observed on day + 9 after CAR T-cell infusion. - Transient increase of transaminases (spontaneously reversible)	- Anti-GAD65 titers in serum decreased from 1:3.200 at baseline to 1:1.000 at day + 56 and to 1:320 by day + 144. - B-cell levels were low following previous immunotherapy and remained low

Results from ClinicalTrials.gov: CAR T cells + multiple sclerosis: 7 results, CAR T cells + NMOSD = 5 results (3 duplicates), CAR T cells + MOG = 1 result (duplicate), and CAR T cells + autoimmune encephalitis: 1 result (duplicate)

*MS* multiple sclerosis, *RRMS* relapsing–remitting MS, *MG* myasthenia gravis, *NMOSD* neuromyelitis optica spectrum disorders, *MOGAD* myelin oligodendrocyte glycoprotein antibody-associated disease, *SPS* stiff-person syndrome, *CAR* chimeric antigen receptor, *BCMA* B-cell maturation antigen, *AQP4* Aquaporin-4, *IgG* immunoglobulin G, *CRS* cytokine release syndrome, *ICANS* immune effector cell-associated neurotoxicity syndrome, *CSF* cerebrospinal fluid, *SLE* systemic lupus erythematosus, *CIPD* chronic inflammatory demyelinating polyneuropathy, *IIM* idiopathic inflammatory myopathy, *EDSS* expanded disability status scale, *OCB* oligoclonal bands, *CTCAE* common terminology criteria for adverse events, *AE* adverse events, *DLT* dose-limiting toxicity, *SAE* serious adverse events, *AESIs* adverse events of special interest

^A^NEX-T CAR T cells; a process designed to shorten manufacturing time and improve the potency and phenotypic attributes of the CAR T-cell drug product to enhance depth and durability of response [49]

^a–e^Quality Rating Scheme for Studies and Other Evidence: ^a^Properly powered and conducted randomized clinical trial; systematic review with meta-analysis, ^b^Well-designed controlled trial without randomization; prospective comparative cohort trial, ^c^Case–control studies; retrospective cohort study, ^d^Case series with or without intervention; cross-sectional study, ^e^Opinion of respected authorities; case reports.

Text box 2 Rationale for CAR T-cell therapy in different immune-mediated diseases of the central nervous system **Why CAR T cells in MS?**

Over the past decades, the armamentarium of disease-modifying therapies (DMTs) in MS has substantially increased due to the conceptual progress recognizing the significant pathogenic role of not only T cells but also B cells in MS. Following first evidence for possible efficacy using rituximab in phase II clinical studies for relapsing and progressive forms of MS [5, 6] newer B-cell-depleting monoclonal antibodies directed toward CD20 [7, 9, 61] were approved as DMT and have shown convincing efficacy in reducing inflammatory disease activity on MRI and clinically (relapses) while displaying an acceptable safety profile. Despite these advances, effects on disability progression have been modest. A majority of persons with relapsing MS will develop a progressive form of MS, and a smaller proportion of individuals will primarily manifest with a progressive disease course [62]. Disability progression in MS can occur independently of acute clinical relapses and inflammatory MRI activity [63]. It has been proposed that progressive disease is linked to compartmentalized chronic smoldering neuroinflammation in the CNS that remains undetectable on the conventional MRI and may be inaccessible to therapy with mAbs [64]. Ectopic B-cell lymphoid follicles resembling tertiary lymphoid structures in the CNS may drive this smoldering compartmentalized neuroinflammation [65]. Such B-cell follicles may associate with continuous cytokine [66] and antibody secretion, possibly contributing to the detection of oligoclonal bands (OCBs) restricted to the cerebrospinal fluid (CSF). In a study where 123 patients with secondary progressive MS were examined postmortem, the presence of these structures was associated with increased diffuse meningeal and subpial inflammation, which correlated with cortical demyelination [65]. Age at onset of disease, time to progression, time to wheelchair use, and age at death were significantly different in these cases compared to those without B-cell follicle-like structures. The hypothesis that mAbs are insufficient in targeting tissue-resident B cells is further substantiated by the observation that, despite sustained depletion of B cells in both the periphery and CSF, OCBs continue to persist in the CSF during therapy with mAbs, albeit at lower levels [67]. Optimal clinical management of MS may thus require addressing both acute and smoldering neuroinflammation to prevent disease progression and improve or at least stabilize long-term disability outcomes. This has led to starting clinical studies with novel B-cell-directed therapies such as CNS-penetrant inhibitors targeting Bruton’s tyrosine kinase (BTKI). There are six BTKI currently in phase II or III trials for treating MS, mainly focusing on relapsing, but also in progressive MS [68]. However, already the first compound failed to reach the primary endpoint in a phase III clinical study (NCT04338061). Other innovative alternatives, e.g., modified CD20 mAbs designed to reach the CNS compartment via receptor-mediated transcytosis, are in early phase clinical development (NCT05704361). CAR T cells, considering their ability to migrate into all compartments and achieve deeper B-cell depletion and lasting effects, are a promising novel therapeutic strategy currently being explored for MS.

**Why CAR T cells in NMOSD?**

NMOSD encompasses a group of rare, autoimmune-mediated diseases of the CNS that primarily affect the optic nerves, spinal cord, and brainstem, and to a lesser extend supratentorial brain parenchyma. In most NMOSD patients, AQP4-IgG are detected [69], which play a crucial role in the pathogenesis and serve as a key diagnostic marker. Disability progression in individuals with NMOSD is primarily driven by exacerbations with acute new symptoms from which patients often recover less effectively compared to MS patients. NMOSD patients are at high risk of experiencing additional attacks within the first year after disease onset, making early therapy initiation crucial for the disease course [70]. Currently, four immunotherapies are approved for AQP4-IgG-positive NMOSD: eculizumab (complement protein C5 inhibitor) [71], inebilizumab (anti-CD19 monoclonal antibody) [10], ravulizumab (complement protein C5 inhibitor) [72], and satralizumab (monoclonal anti-IL-6 receptor antibody) [73]. Treatment recommendations for AQP4-IgG-negative NMOSD rely on expert opinions and comprise classical immunosuppressive therapies, such as azathioprine, mycophenolate mofetil, or the mAbs rituximab [74] and tocilizumab [75]. Recent studies have shown that discontinuation of immunosuppressive therapies in AQP4-IgG-positive or -negative NMOSD patients, even if previously stable, is associated with an increased risk of attacks in the following 12 months [76] Thus, treatment discontinuation is currently not recommended. Besides, a subset of patients does not respond sufficiently to current therapies and may suffer from recurrence, severe sequelae, and death, making the "living drug" concept of CAR T-cell therapy promising particularly for preventing severe relapses in patients with NMOSD.

**Why CAR T cells in MOGAD?**

MOGAD is commonly linked with acute disseminated encephalomyelitis, optic neuritis, or transverse myelitis, and less frequently with cerebral cortical encephalitis, brainstem presentations, or cerebellar presentations. The disease can manifest as either a monophasic or relapsing course. Utilizing MOG-IgG cell-based assays is crucial for achieving diagnostic accuracy in MOGAD cases. Disease flares in MOGAD are generally treated with high-dose corticosteroids or a combination of intravenous corticosteroids and plasma exchange/immune adsorption in patients with severe attacks. In relapsing MOGAD, periodic infusions of intravenous immunoglobulin (IVIg), oral corticosteroids (OC), azathioprine, or mycophenolate mofetil may be all considered for maintenance treatment [77]. In contrast to other antibody-mediated conditions, including AQP4-positive NMOSD, the efficacy of rituximab in MOGAD appears to be only partial, around one-third of patients experience relapses despite complete B-cell depletion [78]. In a recent meta-analysis, the combined results for relapse rate and adverse events, and annualized relapse rate and adverse events showed that IVIG and OC were the most effective and tolerable therapies [77]. Small case series suggest that tocilizumab might be effective in patients with MOGAD refractory to other immunosuppressive treatments [79]. Currently, two compounds are in clinical studies to treat MOGAD, namely the IL6-receptor blocker satralizumab (NCT05271409), and the neonatal Fc receptor blocking agent rozanolixizumab (NCT05063162). All current therapies are off-label with limited efficacy and safety data for this indication. Despite being among the most effective treatments, the long-term use of IVIGs and OC is challenging due to high costs, frequent hospital visits, and supply shortages for IVIG, and the risk of severe side effects for OC. Therefore, there is a significant need for alternative long-term therapeutic strategies.

**Why CAR T cells in AE?**

Autoimmune encephalitis (AE) encompasses a diverse group of inflammatory autoimmune disorders affecting the brain parenchyma with potential involvement of the meninges and spinal cord [80]. Suggested mechanisms that may trigger AE include tumors (paraneoplastic) and infections (parainfectious). Only AE with defined autoantibodies targeting surface proteins will be discussed here [4]. Among these, N-methyl-D-aspartate receptor (NMDAR) AE and leucine-rich glioma-inactivated protein 1 (LGI1) AE are the most prevalent, followed by gamma-aminobutyric acid type B receptor (GABABR) AE [80]. The localized accumulation of CD20 + B cells and CD138 + plasma cells observed in brain tissue biopsies from individuals with NMDAR AE suggests that autoantibodies may originate from activated immune cells infiltrating the CNS [81]. During the acute phase of AE, high-dose steroids represent the preferred first-line immunotherapy, followed by a combination of steroids and intravenous immunoglobulins (IVIG) and plasma exchange/immune adsorption. In cases where pulsed steroid regimes prove ineffective, transitioning to second-line immunotherapy is warranted. In NMDAR AE cases, possible benefits of rituximab, cyclophosphamide, bortezomib (proteasome inhibitor), and tocilizumab were claimed in several case studies [80, 82]. Ocrelizumab, ofatumumab, and daratumumab (IgG1 monoclonal antibody toward CD38) [83–85] have only been studied in individual cases or small series. For further AE such as LGI1 or CASPR2 AE, rituximab, ofatumumab, and tocilizumab were applied off-label [80]. The use of daratumumab in CASPR2 AE was associated with serious adverse reactions, including death [83, 84]. Additionally, daratumumab and tocilizumab have shown therapeutic effects in individual case studies on serum and cerebrospinal fluid-negative and no-antibody-specified AE, respectively [80]. A phase III randomized, double-blind placebo-controlled multicenter basket study, currently underway (NCT05503264), aims to assess the efficacy, safety, pharmacokinetics, and pharmacodynamics of satralizumab in patients with NMDAR and LGI1 AE. Inebilizumab (NCT04372615) and Bortezomib (NCT03993262) are both being evaluated for AE in phase IIb, double-blind, randomized-controlled trials that are currently recruiting. At present, there is no approved therapy for AE. Although the aforementioned medications have clinical utility, they are all off-label treatments with insufficiently proven benefits. Some patients may experience progressive disease if not treated early and aggressively, particularly those with LGI1 AE, and other may necessitate extended stays in intensive care units, often required in NMDAR AE. CAR T-cell therapy emerges as a highly promising modality for AE, potentially depleting B cells within the CNS compartment that catalyze the intrathecal synthesis of pathogenic antibodies.

### First experience in applying CAR T cells for treatment of multiple sclerosis

Preclinical data on anti-CD19 CAR T cells in MS are inconsistent. One study explored their use in a spontaneous opticospinal model of EAE that previously showed lack of disease protection with anti-CD20 mAb therapy. The model associates with meningeal B-cell aggregates considered to drive secondary disease progression in human MS. As expected, after the application of anti-CD19 CAR T cells, effective B-cell depletion and sustained reduction of meningeal aggregates were shown. However, in this model, clinical scores worsened. This was explained by the authors by a possible immunomodulatory function of the meningeal aggregates in this specific animal model [42]. Conversely and in response to this publication, another study using a B-cell-dependent induced EAE model found that anti-CD19 CAR T cells ameliorated EAE and effectively depleted B cells in peripheral tissues and the CNS [43]. This study is promising, but the first study exposes that a varying immunopathology in different models of EAE can lead to conflicting results, not allowing firm conclusions with regards to the clinical application in persons with MS.

Nevertheless, clinician scientists from the University Medical Center Hamburg, Germany, have recently reported the first clinical application of CD19 CAR T cells in two patients with progressive forms of MS [44]. The treatment exhibited an acceptable safety profile with stable clinical MS symptoms observed over a 100-day follow-up period. Both patients had previously received ocrelizumab before initiating CAR T-cell therapy. Notably, CAR T-cell expansion in the CSF was observed in both cases without clinical signs of early neurotoxicity. This finding is particularly relevant as CAR T-cell expansion in the CSF has previously been reported only in the context of ICANS in patients with lymphoma [45]. In one case, intrathecal antibody production in the CSF decreased significantly, with CSF-restricted oligoclonal bands (OCBs) reducing from 13 to 6 by day 14 post-infusion, highlighting CAR T-cell therapy's potential to penetrate immune compartments inaccessible to systemic administration of B-cell-depleting mAbs. Conversely, the number of OCBs and intrathecal immunoglobulin levels in the other patient remained unchanged on day 14 of therapy. An ongoing phase 2 clinical trial aims to evaluate anti-CD19 CAR T cells in 120 patients with progressive MS (NCT06384976) and is expected to provide further insights into efficacy and safety. Additionally, a phase I study aims to include 98 individuals with relapsing or progressive MS (NCT06220201, Table 1).

### Neuromyelitis optica spectrum disorder (NMOSD)

An ongoing open-label phase I clinical trial, assessing the safety of BCMA CAR T-cell therapy in 12 patients with refractory AQP4-IgG positive NMOSD, reported first results in January 2023 [46]. CAR T-cell therapy demonstrated a manageable safety profile and promising therapeutic potential over a median follow-up of 5.5 months. All patients exhibited a decrease in the EDSS score, with four patients improving from being restricted to a wheelchair or bed to walking with or without assistance. Additionally, nine patients showed improvement in bowel and bladder function. Eleven patients experienced no relapses, and, overall, reported improvements in disability and quality-of-life measures. AQP4 antibodies in sera of 11 individuals declined in titer levels. However, all patients experienced grade 1–2 CRS and grade 3 or higher adverse events, such as neutropenia, anemia, and thrombocytopenia. No neurologic toxic effects, ICANS, or dose-limiting toxicity were observed. Post-infusion anti-drug antibodies were reported in three patients, with unclear relevance for potential re-exposure. A notable limitation of the study was the unavailability of newly approved therapies (eculizumab/ravulizumab, satralizumab, and inebilizumab) in China at the trial's commencement, i.e., no participants had received these treatments (Table 1).

### MOG antibody-associated disease (MOGAD)

No registered therapies for MOGAD are available so far, and CAR T cells have not yet been investigated. The ongoing early phase I, open-label basket study evaluating the safety and efficacy of BCMA-directed CAR T cells for antibody-associated inflammatory diseases of the CNS, including MOGAD, may provide first insights into the potential role of CAR T cells in treating this condition (Table 1).

### Autoimmune encephalitis

The efficacy and safety of CAR T-cell therapy targeting universal markers in AE in humans have not yet been extensively documented. A recently published case study described the administration of CAR T cells to a patient with treatment-refractory stiff-person syndrome (SPS) with anti-GAD65 (glutamic acid decarboxylase) antibodies in both CSF and serum, which specifically target GABAergic inhibitory pathways. The patient showed remarkable improvement in leg stiffness, daily walking distance, walking speed, pain, and fatigue over a 5-month follow-up period. Additionally, GABAergic medication was reduced by 40%, and no further immunotherapy was required following CAR T-cell infusion [47].

Reincke et al. [40] developed NMDAR-specific chimeric autoantibody receptor T cells (NMDAR CAAR T) and demonstrated through in vitro experiments that NMDAR CAAR T cells were activated and secreted interferon-gamma and granzyme B, leading to specific lysis of target cells even in the presence of high titers of NMDAR autoantibodies. In a passive transfer mouse model with immunodeficient mice lacking natural killer cells and lymphocytes, CAAR T-cell treatment reduced NMDAR autoantibody-producing target cells and eliminated autoantibodies in serum and in the brain without evidence of toxicity or adverse effects. The in vivo experiment only assessed the efficacy and off-target effects of CAAR T within 20 days and did not investigate the duration of its effect [40], nevertheless providing the rationale for initiation of a phase I clinical trial and offering valuable insights for designing CAAR T cells also for other forms of AE and further autoantibody-mediated diseases.

### Current challenges of *CAR* T-cell therapy in neuroimmunological disorders of the CNS

The application of CAR T-cell therapy in patients with autoimmune diseases presents several challenges. Individuals often have a history of glucocorticoid and other immunosuppressive treatments that may negatively impact T-cell quantity and quality, potentially complicating the retrieval of an adequate number of functional T cells. Nevertheless, preliminary data from case series including patients with rheumatic diseases [24–28] suggest that this may not be a significant issue in CNS-directed autoimmunity.

Regarding availability, CAR T cells have the advantage of persisting and self-amplifying in the body, providing sustained effects, whereas mAbs require multiple administrations due to their limited half-life. While lower pricing mAbs such as rituximab are widely available, CAR T cells can only be applied in expert centers. Besides, considering that to date it takes several weeks to produce the individualized cell therapy, the treatment may not be an option for rapidly progressing cases unless bridging therapy is available. To address this limiting factor, local on site-production is needed, and the future development of allogeneic “off-the-shelf” and “universal CAR T cells” is discussed [48].

With regards to a possible target population for the use of CAR T cells in clinical studies, an unmet need can be clearly defined for persons with MS who exhibit (rapid) EDSS progression despite the use of approved high-efficacy disease-modifying therapy. Therefore, the first cases published, most phase I studies (NCT06138132, NCT06451159), and the ongoing phase 2 clinical trial (NCT06384976) focus on persons with progressive forms of MS. However, while in NMOSD, MOGAD, and AE, disease-defining autoantibodies can be monitored, an ideal surrogate biomarker to monitor treatment response is lacking. Monitoring OCBs and conducting CSF analysis—along with MRI and serum neurofilaments—appear important within pivotal clinical trials, especially in progressive forms where MRI-detectable inflammatory activity is scarce. However, to date, it remains unclear if OCB reduction or reversion to negativity associates with clinical response, and the necessity for repeated CSF testing makes this approach challenging.

Another important aspect is that CAR T-cell therapy in MS has so far only been tested in two individuals with progressive MS without high inflammatory activity. In general, persons with progressive forms of MS pre-treated, e.g., with CD20-directed mAbs demonstrate minimal signs of inflammation (no relapses, minimal-to-no MRI inflammatory activity). Added effects on disease progression may be small and readouts, such as the EDSS score, insensitive in capturing relevant changes during the observational period of phase II/III clinical trials. Furthermore, symptoms and further progression may result from permanent organ damage, which limits symptom reversibility and increases the risk of study failure even after successful “reprogramming” of initially causal autoimmunity following the CAR T-cell treatment. Therefore, earlier application of CAR T cells for treating of autoimmune CNS diseases may be crucial to minimize the risk of permanent organ damage. However, the availability of approved and effective immunotherapies complicates the early use of CAR T cells, feasible as experimental therapy only after several registered treatments have failed, particularly in MS and AQP4-positive NMOSD.

The safety profile of CAR T-cell treatment for highly inflammatory, CNS-targeted autoimmune disorders like relapsing MS is still unclear while it is well established for several effective mAbs approved for MS or AQP4-positive NMOSD. Therefore, randomized-controlled studies may first target, e.g., persons with relapsing forms of MS with ongoing inflammatory disease activity (relapses and/or MRI activity) despite anti-CD20 therapy. Follow-up of this patient subpopulation after a single course of CAR T-cell therapy will then be needed to better understand if prolonged remissions with acceptable long-term risks are achievable. If this could be shown, a broader indication in early, also treatment-naive inflammatory forms of MS would be a next possible step. Overall, strong patient involvement appears advisable when designing such clinical studies, particularly in inflammatory active early MS where patients may benefit most. Even though approved mAbs may effectively control inflammatory disease activity, a significant proportion of MS patients is likely to opt for taking considerable risks if a new therapy offers the hope for a cure or at least long-term treatment-free absence of MS-related disease activity and progression.

## Conclusion

CAR T-cell therapy holds promise as a novel treatment avenue for refractory and relapsing autoimmune CNS diseases where discontinuation of treatment is not recommended. The "living drug" concept underlying CAR T-cell therapy offers the potential for preventing severe relapses. Its migratory capability within the CNS compartment allows for more effective depletion of B cells responsible for the intrathecal synthesis of pathogenic autoantibodies. Currently, the data on the application in individuals with CNS-directed autoimmunity are scarce, and class 1 evidence for efficacy is lacking. However, the available evidence suggests that CAR T-cell therapy is feasible, well-tolerated, and comes with the promise of longer treatment-free remissions and a potential reboot of the B-cell compartment. Further multicenter clinical trials with larger sample sizes are warranted to assess its clinical efficacy, safety, long-term effects, the optimal design, including lymphoablative therapy, dosing, technical demands, and costs. Additionally, identifying the optimal targets for CAR T cells in autoimmune CNS diseases with multiple antigens remains a challenge. Given that B-cell-directed mAbs have demonstrated efficacy in several autoimmune CNS diseases, investigator-initiated and industry-funded basket studies appear to be a reasonable approach to generating higher quality data for treatment-refractory MS or AQP4-positive NMOSD, and for individuals with MOGAD and AE who choose to participate in a study rather than receiving off-label therapy. The prospect of antibody-specific cell depletion with CAAR T cells without associated extensive B-cell depletion raises hope for even more promising therapies.

## Supplementary Information

Below is the link to the electronic supplementary material.

Supplementary figure 1. PRISMA flow diagram of identification, screening, eligibility assessment, and inclusion of studies. Supplementary file1 (PDF 12 KB)

Supplementary figure 2. Transient mRNA-based CAR T cell vs. stable CAR T cell therapy; the CAR-encoding mRNA does not replicate together with the activated and proliferating CAR T cells, so the number of CAR+ cells is determined and limited by the administered dose and declines over time, potentially enabling more precise pharmacokinetic control. The mRNA CAR-activity is however restricted and repeated administration appears necessary. Supplementary table 1. B cell differentiation stages and their cell surface antigens. *long-lived plasma cells in the bone marrow can either be CD19+ or CD19 negative. Supplementary file2 (PDF 149 KB)

Supplementary Table 1. PB cell differentiation stages and their cell surface antigens. *long-lived plasma cells in the bone marrow can either be CD19+ or CD19 negative. Supplementary Table 1 (DOCX 14 KB)

## Abbreviations

CAR: Chimeric antigen receptor
RA: Rheumatoid arthritis
SLE: Systemic lupus erythematosus
MS: Multiple sclerosis
MOGAD: Myelin oligodendrocyte glycoprotein antibody disease
mAbs: Monoclonal antibodies
NMOSD: Neuromyelitis optica spectrum disease
CNS: Central nervous system
BCMA: B-cell maturation antigen
ICANS: Immune effector cell-associated neurotoxicity syndrome
CRS: Cytokine release syndrome
AChR: Acetylcholine receptor
MuSk: Muscle-specific kinase receptor
IgG: Immunoglobulin G
NMDAR: N-methyl-D-aspartate receptor
AE: Autoimmune encephalitis
SPS: Stiff-person syndrome
OCBs: Oligoclonal bands
CAAR T: Chimeric autoantibody receptor T
IVIG: Intravenous immunoglobulins
NMDAR: N-methyl-D-aspartate receptor
LGI1: Leucine-rich glioma-inactivated protein 1
GABABR: Gamma-aminobutyric acid type B receptor
EDSS: Expanded disability status scale
BTKI: Bruton tyrosine kinase inhibitor
DMT: Disease-modifying therapy
