# Supplementary figures and images for: Chimeric antigen receptor T-cell therapy for autoimmune diseases of the central nervous system: a systematic literature review
Source: J Neurol. 2024 Sep 14;271(10):6526–42. doi: 10.1007/s00415-024-12642-4 (PMC11446985; doi:10.1007/s00415-024-12642-4)

Identification of studies via databases and registers

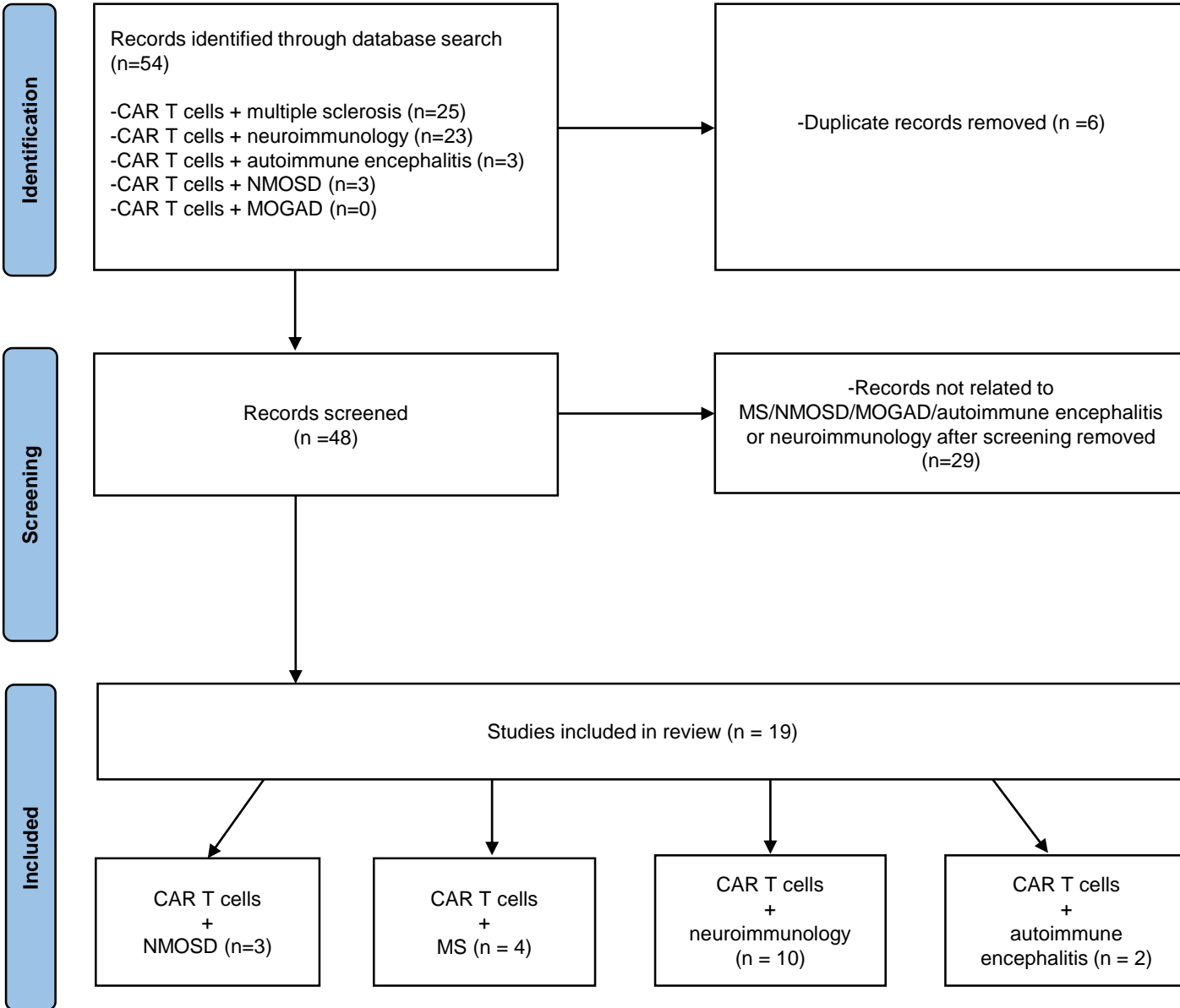

Supplement: Supplementary file 1 — Supplementary figure 1. PRISMA flow diagram of identification, screening, eligibility assessment, and inclusion of studies. Supplementary file1 (PDF 12 KB) [file 415_2024_12642_MOESM1_ESM.pdf]

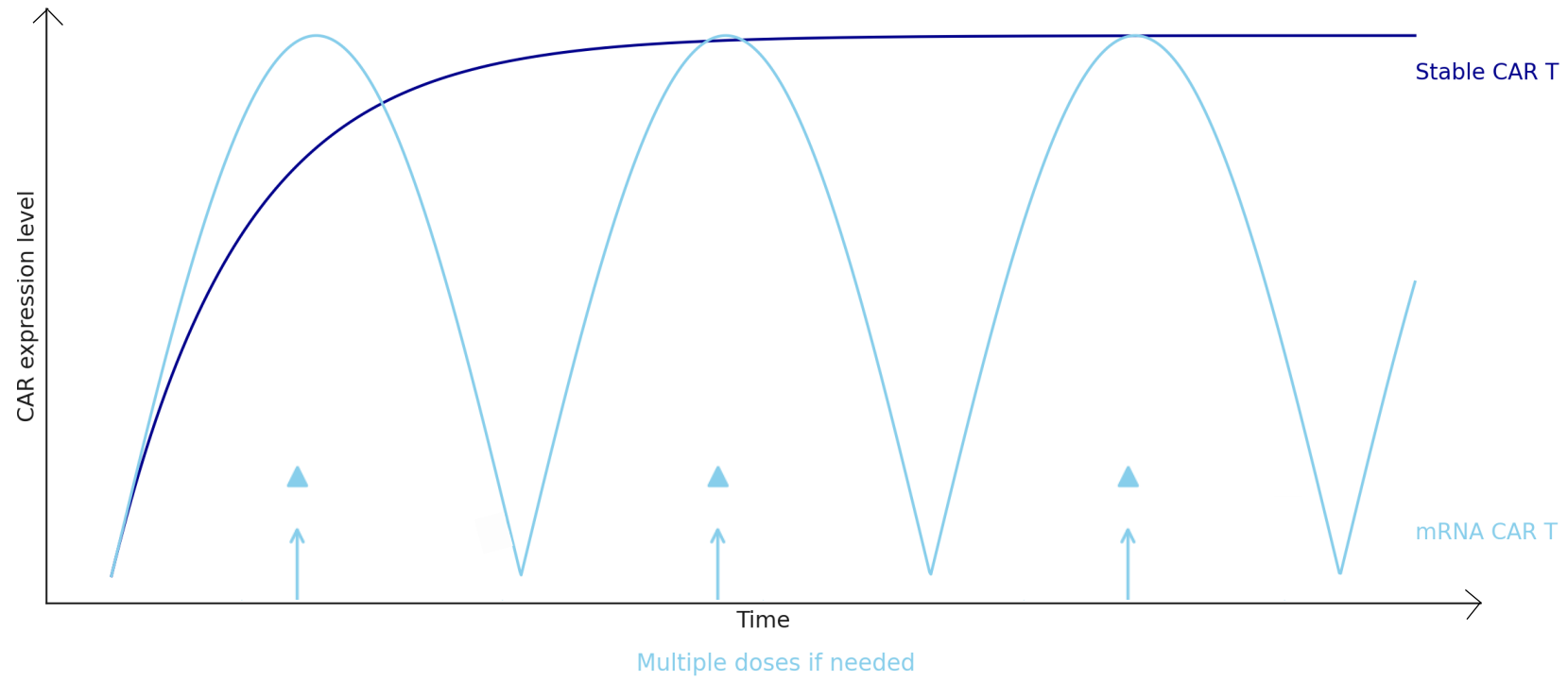

Supplement: Supplementary file 2 — Supplementary figure 2. Transient mRNA-based CAR T cell vs. stable CAR T cell therapy; the CAR-encoding mRNA does not replicate together with the activated and proliferating CAR T cells, so the number of CAR+ cells is determined and limited by the administered dose and declines over time, potentially enabling more precise pharmacokinetic control. The mRNA CAR-activity is however restricted and repeated administration appears necessary. Supplementary table 1. B cell differentiation stages and their cell surface antigens. *long-lived plasma cells in the bone marrow can either be CD19+ or CD19 negative. Supplementary file2 (PDF 149 KB) [file 415_2024_12642_MOESM2_ESM.pdf]
