# Supplementary material for: Chimeric antigen receptor T-cell therapy for autoimmune diseases of the central nervous system: a systematic literature review
Source: J Neurol. 2024 Sep 14;271(10):6526–42. doi: 10.1007/s00415-024-12642-4 (PMC11446985; doi:10.1007/s00415-024-12642-4)
Supplement: Supplementary file 3 — Supplementary Table 1. PB cell differentiation stages and their cell surface antigens. *long-lived plasma cells in the bone marrow can either be CD19+ or CD19 negative. Supplementary Table 1 (DOCX 14 KB) [file 415_2024_12642_MOESM3_ESM.docx]

|  | Bone marrow | | | Periphery and lymph node or tertiary lymphoid tissue | | | Bone marrow |
| --- | --- | --- | --- | --- | --- | --- | --- |
| Cell Surface antigen | Pro B cell | Pre B cell | Immature B cell | Mature B cell | Memory B cell | Plasmablast | Plasma cell |
| CD19 | x | x | x | x | x | x | x* |
| CD20 |  | x | x | x | x |  |  |
| CD22 |  | x | x | x | x |  |  |
| BCMA |  |  |  |  | x | x | x |
| CD38 |  |  |  |  | x | x | x |
| CD126 |  |  |  |  | x | x | x |
| CD138 |  |  |  |  |  |  | x |

## Supplementary table 1. B cell differentiation stages and their cell surface antigens. *long-lived plasma cells in the bone marrow can either be CD19+ or CD19 negative.
